# Incidence and Risk Factors for Dysphagia Following Cerebellar Stroke: a Retrospective Cohort Study

**DOI:** 10.1007/s12311-023-01564-y

**Published:** 2023-05-19

**Authors:** Li Huang, Yunlu Wang, Jikang Sun, Lequn Zhu, Jimin Liu, Yuwei Wu, Chunlei Shan, Juntao Yan, Ping Wan

**Affiliations:** 1 Yueyang Hospital of Integrated Traditional Chinese and Western Medicine, Shanghai University of Traditional Chinese Medicine, 110 Ganhe Road, Hongkou District, Shanghai, 200437 China; 2 Shanghai University of Traditional Chinese Medicine, 1200 Cailun Road, Pudong New District, Shanghai, 201203 China

**Keywords:** Dysphagia, Cerebellar stroke, Incidence, Rate, Prognosis

## Abstract

The cerebellum is known to play a supportive role in swallowing-related functions; however, wide discrepancies about the incidence rate of swallowing disorders following cerebellar strokes exist within the literature. This study aimed to investigate the incidence rate of dysphagia and the factors which may affect the presence of dysphagia and clinical recovery in individuals diagnosed with cerebellar stroke. A retrospective chart audit of 1651 post-stroke patients (1049 males and 602 females) admitted with a cerebellar stroke to a comprehensive tertiary hospital in China was conducted. Data on demographics, medical, along with swallowing function assessment were collected. Differences between dysphagic and non-dysphagic groups were evaluated using *t*-tests and Pearson’s chi-square test. Univariate logistic regression analysis was performed to establish factors associated with the presence of dysphagia. A total of 11.45% of participants were identified with dysphagia during inpatient admission. Individuals with mixed types of stroke, multiple lesions in the cerebellum, and ages older than 85 years old were more likely to develop dysphagia. Moreover, the prognosis of dysphagia following a cerebellar stroke was associated with lesions in different parts of the cerebellum. The cumulative recovery rates from the best to worse were the right hemisphere group, the cerebellum vermis or peduncle group, and both the hemisphere group and the left hemisphere group, respectively.

## Introduction

The cerebellum, located in the lower portion of the brain and occupies about one-eighth of the intracranial space, has less than 10% stroke incidence among all strokes [1, 2]. Approximately, 2–3% of all ischemic strokes and 10–15% of all intracerebral hemorrhages occur in the cerebellum [1, 3]. Several studies reported that lesions in the cerebellum did not contribute to the presence of dysphagia [4–8]. However, one cohort study reported that seven of the eleven subjects presenting with a lesion to the cerebellum exhibited aspiration in the swallow evaluation [9]. Previous studies also reported that pathology affecting the cerebellum could impair swallowing physiology in both the oral and pharyngeal phases [10–12]. Meanwhile, findings of the cerebellum in controlling swallowing in studies using brain imaging techniques are also not consistent. Some studies demonstrated that the cerebellum participated in the control of swallowing [13–17], and other studies reported that the cerebellum was not activated during the deglutition process [18, 19].

Recent evidence support that the cerebellum acts to modulate the cerebral motor cortical areas and ensure accurate, smooth, and coordinated muscular activity [10]. The cerebellum has recently become a focus of interest in treating dysphagia in the field of neuromodulation [20, 21]. Evidence suggested that cerebellar repetitive transcranial magnetic stimulation (rTMS) could reverse the suppressed activity in the cortical swallowing system, facilitate corticobulbar pathways to the pharynx, and restore swallowing function in healthy adults [22, 23]. Moreover, evidence revealed that cerebellar transcranial magnetic stimulation (TMS) could intensify cortical firing for swallowing and evoke motor responses in the pharynx for patients with dysphagia [15, 20, 22, 24]. Swallow function was modulated through rTMS/TMS indicating that the cerebellum has connections with cortical, subcortical, brainstem, and peripheral structures, through which the cerebellum may regulate the neuron related to swallowing and organize the coordinated sequence of muscle activity during the swallowing process [10, 13, 15, 16, 25]. This evidence supports the theory that the cerebellum may complement cortical and brainstem control for swallowing, intensifying neural responses, and coordinating selective attention to aid the execution of generated in the cortex, and plays an important role in feed-forward mechanisms, and controlling timing, sequencing, feedback, and internal coordination for sensory input and motor outputs of oral-lingual and pharyngeal musculature during deglutition [10, 15, 26]. The role of the cerebellum in deglutition has become increasingly evident.

Nevertheless, the data focused on cerebellar lesions is limited, and the exact nature of this role is still under-explored. Lesions in the cerebellum were testified to disrupt the motor modulatory influence on cerebral motor cortical areas and develop swallowing disorders [27, 28]. However, the incidence of dysphagia after a cerebellar stroke reported in the previous literature varies greatly from 0 to 63%. And the risk factors for dysphagia, such as demographic characteristics, lesioned locations, ischemic or hemorrhagic stroke, and the evolution of dysphagia, are still not fully understood [5, 15, 29]. Although studies demonstrated that patients with cerebellar stroke had favorable outcomes, those patients with dysphagia would have less favorable outcomes, increased mortality, and a longer stay in healthcare facilities with higher institutionalization rates at 3 months post-stroke [30–32]. During decades of clinical practice and research on people with dysphagia, the authors’ team also found that some cases with pure lesions in the cerebellum could relieve swallowing difficulties while others with pure lesions in the cerebellum had less benefit from the existing intervention for dysphagia and had a poor prognosis(included in this retrospective study), which drew our attention of the potential relationship between the dysphagia and the lesions in the cerebellum (i.e., right/left cerebellum, bilateral cerebellum, vermis, and peduncle).

Therefore, this study aims to investigate the incidence of dysphagia and risk factors potentially influencing the risk of developing dysphagia after a cerebellar stroke from a retrospective cohort. We preliminarily speculated that the cerebellum had a role in the regulation of swallowing function, and there might exist a dominant region of swallowing function in the cerebellum, which once is damaged, dysphagia might be irrecoverable.

## Patients and Methods

### Research Design

A retrospective chart review of the medical records of post-stroke patients who were hospitalized in Shanghai Yueyang Hospital of Integrated Traditional Chinese and Western Medicine Affiliated to Shanghai University of Traditional Chinese Medicine (YY Hospital) in China between January 2014 and May 2021 (8-year period) was conducted. Post-stroke patients admitted to YY Hospital involve those who have an acute onset of stroke, look for rehabilitation service, and are readmitted for promoting health with integrated western and traditional Chinese medicine. The study was approved by the medical ethics committee of YY Hospital.

### Study Population

The World Health Organization ICD-10 diagnostic code “I63.904-cerebellar infarction” or “I61.401-cerebellar hemorrhage,” in addition to CT or MRI reports demonstrating lesions in the cerebellum were used to identify all appropriate cases in the digital medical records system. The usual length of stay for all post-stroke patients is about 14 days according to local medical insurance regulations. A total of 2673 cases were identified and were further screened by two researchers. A binary rating of “+/−” was used to identify dysphagia (“+”) or no dysphagia (“−”) resulting from the lesions in the cerebellum.

The inclusion criteria of dysphagia resulting from the lesions in the cerebellum (“+”) were as follows: (1) the first onset of a cerebellar stroke, and dysphagia was reported after assessing with the videofluoroscopic swallowing study (VFSS) and/or the fiberoptic endoscopic evaluation of swallowing (FEES) by a speech-language pathologist (SLP) after admission; (2) the first onset of a cerebellar stroke, with stroke history but without lesions in the cerebellum and without dysphagia records in the medical record (the results of the previous Kubota water swallowing test were grade 1) [33, 34], and dysphagia was reported in the acute phase after assessing with VFSS and/or FEES by a SLP after admission; (3) in the subacute or chronic stage of a cerebellar stroke with the first onset, with dysphagia records during the previous hospitalization in other hospitals (the results of the previous Kubota water swallowing test ranged from grades 3 to 5, and the patient wore a nasogastric tube on admission), and dysphagia was reported after assessing with VFSS and/or FEES by a SLP after current admission.

The inclusion criteria of no dysphagia resulting from the lesions in the cerebellum (“−”) were as follows: (1) the first onset of a cerebellar stroke and no dysphagia (grade 1) was reported through the Kubota water swallowing test by a physician; (2) the first onset of a cerebellar stroke, with stroke history but without lesions in the cerebellum and without dysphagia records in the medical record (the results of the previous Kubota water swallowing test were grade 1), and no dysphagia was reported through the Kubota water swallowing test by a physician; (3) in the subacute or chronic stage of a cerebellar stroke with the first onset, without dysphagia records during the previous hospitalization in other hospitals (the results of the previous Kubota water swallowing test were grade 1), and no dysphagia was reported through the Kubota water swallowing test by a physician.

The exclusion criteria were as follows: (1) the presence of swallowing difficulties before the first onset of cerebellar stroke; (2) the first onset of a cerebellar stroke, but with other lesion sites outside the cerebellum; (3) unable to receive bedside swallowing examination by an SLP due to severe physical and cognitive impairment; (4) repeated admission. A total of 1651 participants were finally eligible for inclusion in the data analysis.

### Data Collection

Data abstraction was conducted according to the methodological guidelines to promote the internal validity and reproducibility of the study [35]. Two reviewers reviewed all of the medical charts independently to ensure consistency of data collection and ensure validity and reliability [36]. Due to the complexity of some data collection and analysis, a discussion team including a physician, a radiologist, and an SLP was formed to make the final decision.

Data obtained in this study included age, gender, type of stroke (i.e., ischemic or hemorrhage), lesion sites in the cerebellum, date of the first onset of a cerebellar stroke and discharge from hospital of that hospitalization, results of swallowing function assessments at the first onset of a cerebellar stroke, and discharge from hospital of that hospitalization. To learn the prognosis of dysphagia following cerebellar strokes, the research team made a follow-up for all the participants who were recorded with dysphagia following the cerebellar stroke by telephone and inquired about their current swallowing function status from September 2021 to November 2021. Therefore, data were collected at three points: the first onset of cerebellar stroke, discharge from YY hospital, and follow-up time by telephone. In order to show the prognosis of dysphagia more intuitively, the statistician artificially divided the follow-up time into every 10 months from the first onset of dysphagia to follow-up time.

### Data Analysis

All collected data were entered into a Microsoft Excel spreadsheet. Descriptive statistics were used to establish the mean (standard deviation [SD]) for continuous variables with normal distribution or median (interquartile range [IQR]) for those with non-normal distribution. Categorical variables were reported as a frequency (percentage). The two groups (dysphagia at discharge vs. dysphagia at follow-up point) were compared using Pearson’s chi-square test for binary variables. Due to the retrospective nature of this study, data for all variables were not available. When sufficient detail was lacking or data were unreported, a percentage of “lost” was reported. Logistic regression analysis was performed to evaluate predictive risk factors with a favorable report of dysphagia as the primary outcome variable. The possible risk factors included age, gender (male/female), type of stroke, and lesion locations in the cerebellum. The results of logistic regression analyses were recorded as odds ratio (OR), 95% confidence interval (CI), and *p*-value. Cumulative recovery of dysphagia in different risk factors groups from the final follow-up point back to the first onset point was visually depicted by survival curves. We performed all analyses using the SPSS software version 25.0, and two-sided statistical significance was defined as *p* < 0.05.

## Results

A total of 1651 post-stroke individuals with lesions in the cerebellum were enrolled in this study, including 1049 male participants and 602 female participants with a mean age of 73.10 (SD = 11.61) years. The majority type of stroke was ischemic stroke (*n* = 1535, 92.97%). The most to least lesions in the cerebellum were the right cerebellum alone (*n* = 599, 36.28%), the left cerebellum alone (*n* = 540, 32.71%), both the right and left cerebellum (*n* = 382, 23.14%), and cerebellar vermis or peduncle (*n* = 26, 1.57%). There was a significant difference between the male and female groups based on age (*p* < 0.05) and none for all other variables. Table 1 summarizes the baseline characteristics of the study population.

**Table 1:** The demographic feature among patients with stroke in Yueyang Hospital of Shanghai, China

Variables	Total patients (*n* = 1651)	Male patients (*n* = 1049)	Female patients (*n* = 602)
Age (years)* (mean, SD)	73.10 (11.61)	70.92 (11.48)	76.89 (10.84)
Age (years)* (median, IQR)	74.00 (65.00, 83.00)	71.00 (63.00, 80.00)	79.00 (70.00, 85.00)
Age groups (years)*, *n* (%)			
Less than 65	401 (24.29)	318 (30.31)	83 (13.79)
65–75	455 (27.56)	313 (29.84)	142 (23.59)
76–85	498 (30.16)	286 (27.26)	212 (35.22)
Over 85	297 (17.99)	132 (12.58)	165 (27.41)
Type of stroke, *n* (%)			
Ischemic stroke	1535 (92.97)	969 (92.37)	566 (94.02)
Hemorrhagic stroke	83 (5.03)	60 (5.72)	23 (3.82)
Mixed stroke	33 (2.00)	20 (1.91)	13 (2.16)
Lesion location of the stroke, *n* (%)			
Left cerebellum	540 (32.71)	352 (33.56)	188 (31.23)
Right cerebellum	599 (36.28)	371 (35.37)	228 (37.87)
Left and right cerebellum	382 (23.14)	238 (22.69)	144 (23.92)
Cerebellar vermis and peduncle	26 (1.57)	15 (1.43)	11 (1.83)
Mixed (at least 3 lesion locations)	104 (6.30)	73 (6.96)	31 (5.15)
Dysphagia induced by cerebellar injury, *n* (%)			
Yes	189 (11.45)	116 (11.06)	73 (12.13)
No	1462 (88.55)	933 (88.94)	529 (87.87)
Dysphagia at discharge, *n* (%)			
Yes	128 (67.72)	77 (66.38)	51 (69.86)
No	43 (22.75)	27 (23.28)	16 (21.92)
Lost to follow-up	18 (9.52)	12 (10.34)	6 (8.22)

*SD* standard deviation, *IQR* interquartile range

*The differences between male and female patients on demographic feature were statistically significant (*P* < 0.05)

### Incidence of Dysphagia Following a Cerebellar Stroke

The overall incidence of dysphagia induced by a cerebellar stroke was 11.45% (*n* = 189), and the incidence of that in males and females was 11.06% (*n* = 116) and 12.13% (*n* = 73), respectively. At the point of discharge from the hospital, 67.72% (*n* = 128) of overall participants with dysphagia during admission reported remained dysphagia, 22.75% (*n* = 43) of these patients reported recovery from dysphagia, and 9.52% (*n* = 18) of these patients’ relevant information of dysphagia could not be found (see Table 1).

### Factors Potentially Influencing the Risk of Developing Dysphagia Following a Cerebellar Stroke

Ischemic and/or hemorrhagic strokes, lesion location in the cerebellum, and age were risk factors for dysphagia in the univariate model. Patients with mixed stroke (OR = 3.00, 95% CI: 1.02–8.83) and mixed (at least 3) lesion locations (OR = 2.16, 95% CI: 1.19–3.90) were associated with higher odds ratios of dysphagia. Moreover, patients with ages older than 85 (OR = 2.53, 95% CI: 1.58–4.07) had a significantly higher odds ratio for dysphagia compared to other age groups (Table 2).

**Table 2:** The dysphagia induced by cerebellar injury among patients with stroke in Yueyang Hospital of Shanghai, China

Variables	Percentage of dysphagia in stroke patients, *n* (%)	Uni-variate logistic regression	
		OR	95% CI
Type of stroke, *n* (%)			
Hemorrhagic stroke	8 (9.64)	1.00	-
Ischemic stroke	173 (11.27)	1.19	0.57–2.51
Mixed stroke*	8 (24.24)	3.00	1.02–8.83
Lesion location of the stroke, *n* (%)			
Left and right cerebellum	38 (9.95)	1.00	-
Left cerebellum	64 (11.85)	1.22	0.80–1.86
Right cerebellum	63 (10.52)	1.06	0.69–1.63
Cerebellar vermis and peduncle	4 (15.38)	1.65	0.54–5.03
Mixed (at least 3 lesion locations)*	20 (19.23)	2.16	1.19–3.90
Sex, *n* (%)			
Male	116 (11.06)	0.90	0.66–1.23
Female	73 (12.13)	1.00	-
Age groups (years)*, *n* (%)			
Less than 65	31 (7.73)	1.00	-
65–75	52 (11.43)	1.54	0.97–2.46
76–85	54 (10.84)	1.45	0.91–2.31
Over 85	52 (17.51)	2.53	1.58–4.07

*OR* odds ratio, *CI* confidence interval

*The differences between stroke patients with and without dysphagia were statistically significant (*P* < 0.05)

### Recovery of Dysphagia Following Stroke with Lesions in the Cerebellum

**Fig. 1:**
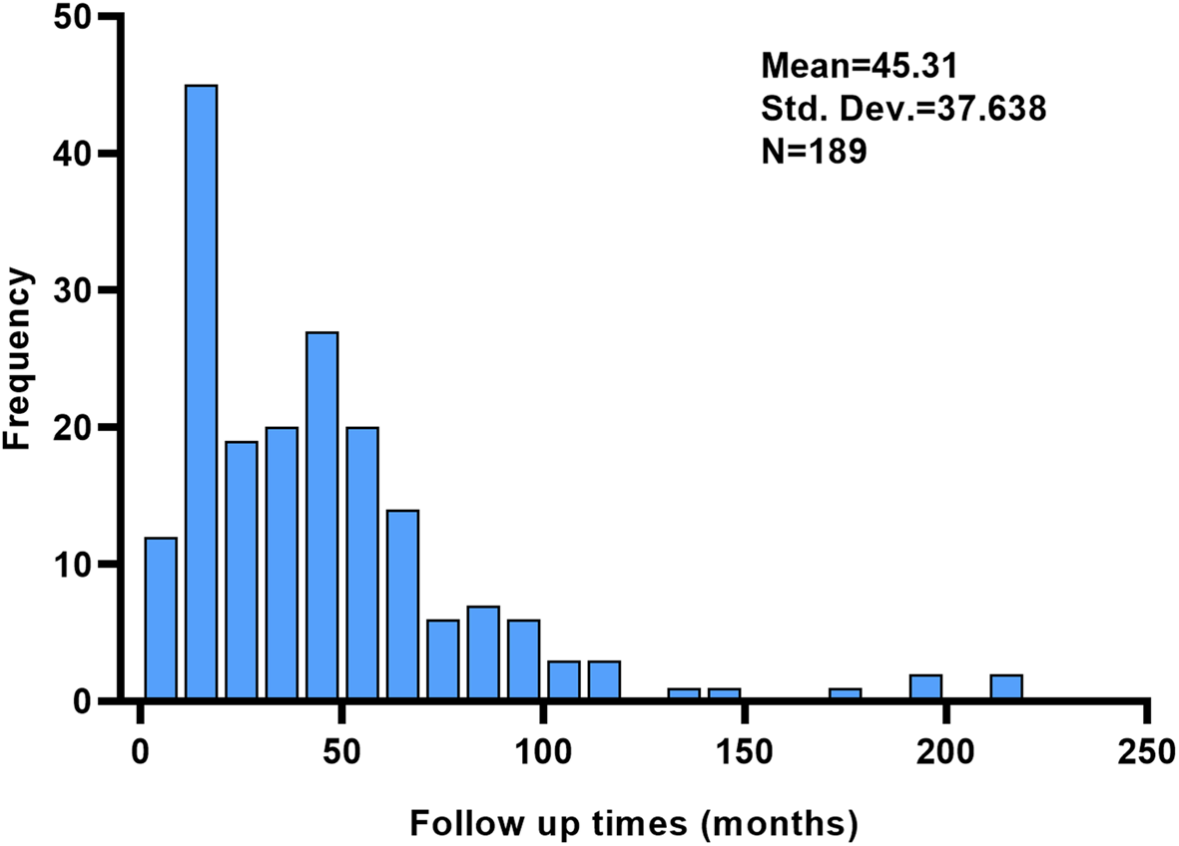
The frequency of patients with dysphagia (*n* = 189) in follow-up times. The mean follow-up period of patients with dysphagia was 45.31 months (standard deviation = 37.638)

Table 3 shows the dysphagia condition of participants during admission (*n* = 189) at the point of hospital discharge and follow-up time. Dysphagia conditions in different lesions in the cerebellum at the point of hospital discharge were significantly different, and so were the dysphagia conditions at different ages at the point of follow-up. Other variables at the two points had no statistical difference (Table 3). Figure 1 demonstrates the case frequency of patients with dysphagia (*n* = 189) every 10-month follow-up time. Looking back from November 2021, a greater number of case frequencies occurred in the closer follow-up time (mean = 45.31, SD = 37.64), and fewer case frequencies with the increased follow-up time.

**Table 3:** The dysphagia condition among stroke patients at different follow-up times in Yueyang Hospital of Shanghai, China (*n* = 189)

Variables	Dysphagia at discharge			Dysphagia at the time of follow-up in the year 2021		
	Lost	Yes	No	Lost	Yes	No
Sex, *n* (%)						
Male	12 (10.34)	77 (66.38)	27 (23.28)	66 (57.39)	33 (28.70)	16 (13.91)
Female	6 (8.22)	51 (69.86)	16 (21.92)	51 (69.86)	15 (20.55)	7 (9.59)
Age groups (years)*, *n* (%)						
Less than 65	1 (3.23)	20 (64.52)	10 (32.26)	12 (40.00)	11 (36.67)	7 (23.33)
65–75	5 (9.62)	35 (67.31)	12 (23.08)	30 (57.69)	13 (25.00)	9 (17.31)
76–85	8 (14.81)	37 (68.52)	9 (16.67)	32 (59.26)	16 (29.63)	6 (11.11)
Over 85	4 (7.69)	36 (69.23)	12 (23.08)	43 (82.69)	8 (15.38)	1 (1.92)
Type of stroke, *n* (%)						
Ischemic stroke	18 (10.40)	113 (65.32)	42 (24.28)	110 (63.95)	41 (23.84)	21 (12.21)
Hemorrhagic stroke	0 (0.00)	7 (87.50)	1 (12.50)	2 (25.00)	5 (62.50)	1 (12.50)
Mixed stroke	0 (0.00)	8 (100.00)	0 (0.00)	5 (62.50)	2 (25.00)	1 (12.50)
Lesion location of stroke**, *n* (%)						
Left cerebellum	5 (7.81)	45 (70.31)	14 (21.88)	38 (60.32)	19 (30.16)	6 (9.52)
Right cerebellum	3 (4.76)	38 (60.32)	22 (34.92)	41 (65.08)	10 (15.87)	12 (19.05)
Left and right cerebellum	7 (18.42)	26 (68.42)	5 (13.16)	24 (63.16)	11 (28.95)	3 (7.89)
Cerebellar vermis and peduncle	0 (0.00)	3 (75.00)	1 (25.00)	1 (25.00)	2 (50.00)	1 (25.00)
Mixed (at least 3 lesion locations)	3 (15.00)	16 (80.00)	1 (5.00)	13 (65.00)	6 (30.00)	1 (5.00)

**The differences at discharge were statistically significant (*P* < 0.05)

*The differences in follow-up in the year 2021 were statistically significant (*P* < 0.05)

**Fig. 2:**
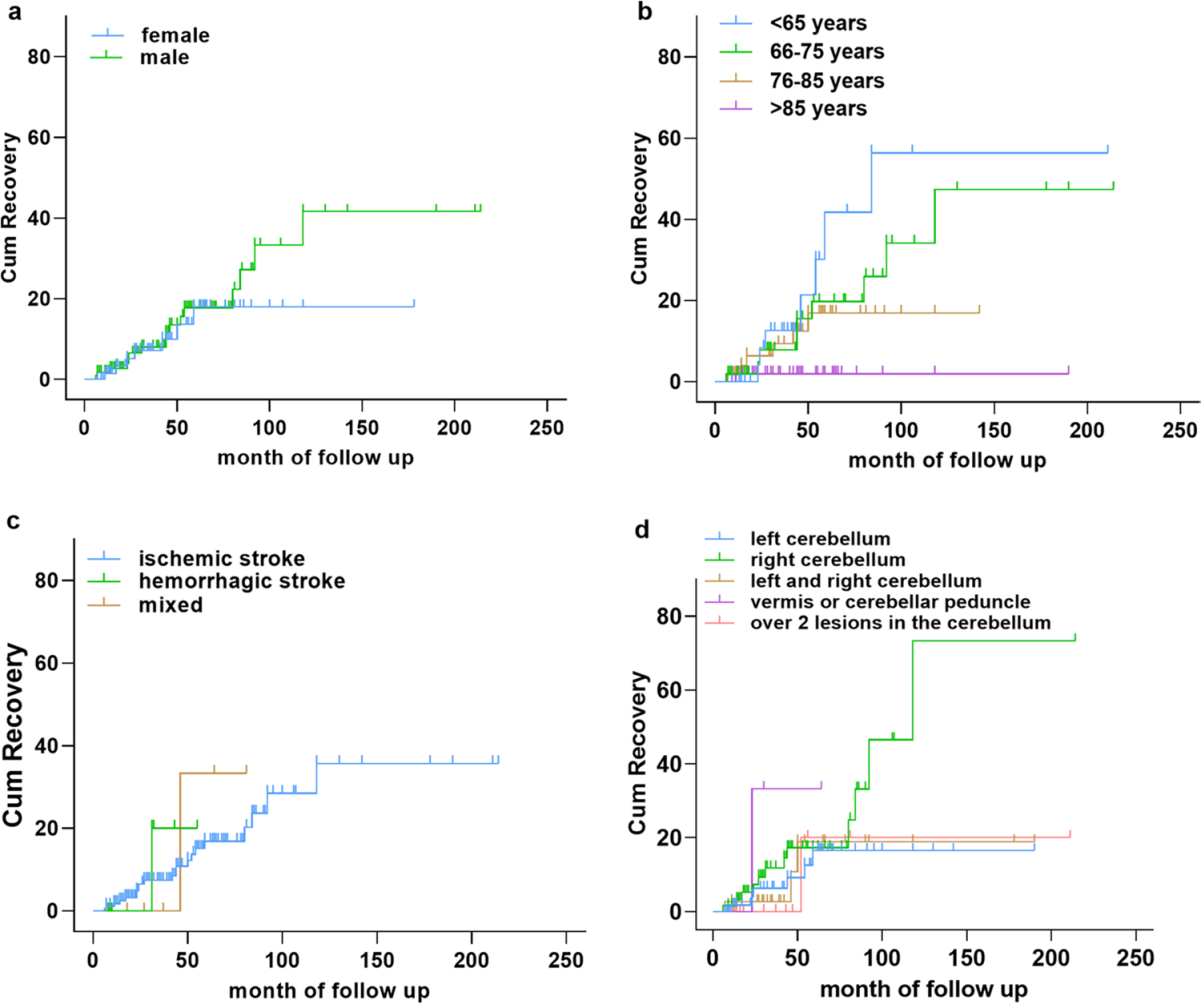
Factors influencing the cumulative recovery of dysphagia following cerebellar stroke. **a** The influence of gender on cumulative recovery of dysphagia, **b** the influence of age on cumulative recovery of dysphagia, **c** the influence of type of stroke on cumulative recovery of dysphagia, and **d** the influence of lesion location in the cerebellum on cumulative recovery of dysphagia

ROC analysis was completed to evaluate the recovery trend of dysphagia resulting from a cerebellar stroke. Four curves represented the influence of age, gender, ischemic and/or hemorrhagic strokes, and lesions in the cerebellum on the recovery of dysphagia. The overall cumulative recovery rate of dysphagia due to a cerebellar stroke in the male group is higher than that in the female group. As the age increased, the cumulative recovery rate became lower. Patients in the hemorrhagic group recovered quicker than those in the ischemic and mixed group, and the number of patients who recovered from dysphagia was greater than that in the ischemic and mixed group. The cumulative recovery rates from the best to worse were the right hemisphere group, the cerebellum vermis or peduncle group, and both the hemisphere group and the left hemisphere group, respectively (Fig. 2).

## Discussion

A growing body of literature has investigated the incidence of dysphagia in people with cerebellar strokes and the role of the cerebellum in swallowing. As previously mentioned, the findings from such studies were inconsistent [6, 8, 11, 12, 37]. This study aimed at exploring whether isolated lesions in the cerebellum were related to the occurrence of dysphagia post-stroke by studying the incidence rate of dysphagia following cerebellar stroke retrospectively. Moreover, this study aimed at exploring the factors that were related to the development and prognosis of dysphagia following cerebellar injuries by analyzing the demographic features of people who had dysphagia post cerebellar strokes. These findings may provide evidence for future clinical practice of swallowing screening and assessment for people who suffer from cerebellar strokes, and research on the role of the cerebellum in the swallowing process.

### Isolated Injuries in the Cerebellum and the Occurrence of Dysphagia

The incidence rate of dysphagia identified in this study was 11.45%, which might imply that the cerebellum was involved in the swallowing process. Our results corresponded with the evidence that the cerebellum was not a part of the primary motor swallowing pathway, but it took part in several circuit loops within the brain, including cerebrocerebellar loops and cerebellospinal loops to fine-tune distantly initiated motor activity [38], so that isolated damage to the cerebellum might cause an element of incoordination of swallowing muscular activity [26, 27, 37, 39]. Therefore, people with cerebellar strokes need to be timely referred to SLPs for a swallowing assessment.

However, lesions in different parts of the cerebellum did not show any statistical difference in developing dysphagia in this study, which might imply that the representations of swallowing musculature occurred in multiple locations over the cerebellum, and both hemispheres of the cerebellum, the vermis and peduncles, might be involved in the swallowing process [11, 12, 27, 37]. Previous studies reported that TMS stimulation at the bi-hemispheric cerebellum was more effective than unilateral cerebellar stimulation to increase pharyngeal motor activities, which supported the evidence that both cerebellar hemispheres could send impulses to contralateral cortical motor areas via dentate nuclei through the superior cerebellar peduncle [27, 28, 40, 41], and fastigial nuclei in each cerebellar hemisphere could send projections to the central pattern generator and then connect to the higher swallowing centers in the cortex [25, 27, 42]. There was also evidence suggesting that the midline vermis, which connected to both sensorimotor systems and the cerebral cortex, was involved in multisensory and motor processing [38], and pharyngeal motor responses could be adjusted through TMS stimulation [13, 22, 24, 40]. Studies described that three peduncles were a critical structure that connects the cerebellum to the brainstem and the motor nucleus of the cerebral cortex [38, 41]. It was considered that the corticopontocerebellar tract transmitted cortical fibers to the cerebellum via the pontine nucleus, while the cerebellothalamocortical tract transmitted cerebellar efferent neurons to the cerebral cortex via the thalamus [43]. It was speculated that the cerebellum affected the cerebral pharyngeal motor cortex through the cerebral pharyngeal motor cortex-pons-cerebellum-cerebral pharyngeal motor cortex circuit, and then indirectly affected the swallowing function of the cricopharyngeal muscle through the corticobulbar pathway of the brain [41, 43, 44].

### Lesions in the Cerebellum and the Recovery of Dysphagia

Two-thirds of patients with post-stroke dysphagia did not recover swallowing function on discharge from the hospital, and lesions in the cerebellum and the age of the patient were correlated with the presence of dysphagia on discharge and in November 2021, respectively, in our study. This result was similar to a previous finding that 66.29% of patients with dysphagia on admission would discharge with dysphagia [45], and consistent with the suggestion that lesioned locations had a relevant effect on swallowing dysfunction [13, 46]. Moreover, the dysfunction courses for patients with dysphagia (*n* = 189) in our study, tracing back from November 2021 to the first month of dysphagia onset, showed that the mean course of dysphagia was about 3.8 years and the longest course was 20.8 years. The course of most recruited patients was less than the mean course in this study, and there were more patients lost mainly due to different causes of death after stroke in the follow-up point of November 2021(*n* = 117) than on discharge(*n* = 18). This result was similar to the report that the mortality for post-stroke dysphagia ranged from 20 to 37%. Therefore, dysphagia had a large impact on survival and clinical recovery after stroke [47–49].

The recovery rate of the lesions in the cerebellum from best to worst was right cerebellum, vermis or peduncle, bilateral cerebellar hemisphere, and left cerebellum in this study, which might imply that the left cerebellum played a more important role than the right side in controlling swallowing process. Several previous studies have pointed out that isolated lesion in the left cerebellum was more likely to develop swallowing difficulties, such as delayed swallow initiation, impaired laryngeal closure, oral and hypopharyngeal residue, and aspiration [10, 14, 16, 50–52]. In neuroimaging studies of healthy participants, the cerebellar hemispheres and the cerebellar vermis were observed to be activated during the normal swallowing process, with noticeably strong activation in the left cerebellar hemisphere [51, 53]. Moreover, the representation of the lips and tongue involved in swallowing was related to both the cerebellar hemispheres and the cerebellar vermis [51, 53]. Besides, studies of rTMS and transcranial direct current reported that stimulation on the cerebellar vermis and the left cerebellar peduncle was related to reduced swallowing disorders, which suggested that the vermis and the peduncles played roles in controlling swallowing [23, 54]. Our results supported the assumption that the cerebellum had a multi-limbed motor homunculus, with its surface corresponding to different body areas; the same muscle groups were represented in various locations over the cerebellum, and there might exist functional lateralization for swallowing in the cerebellum [10, 23, 27, 53, 55].

### Potential Risk Factors Related to the Development and Recovery of Dysphagia Following Cerebellar Strokes

Multiple lesions in the cerebellum, a mixed type of cerebellar stroke, and ages older than 85 years old were related to a higher possibility of dysphagia in this study. These results are corroborated by previous evidence that cumulative damage to the brain was more likely to cause dysphagia and worsen conditions in post-stroke patients [45, 56, 57], and older age is one of the potential risk factors for swallowing disorders after strokes [58, 59]. Therefore, special attention is needed when assessing swallowing problems in patients of older age and with mixed types of cerebellar stroke [7].

The type of stroke, age, and gender were associated with the different clinical recovery from dysphagia in our retrospective study. The cumulative recovery of the ischemic type was better than that of the hemorrhagic and mixed type in this study, which was not consistent with the previous studies that ischemic strokes were more likely to have a relatively poor recovery than those with hemorrhagic strokes [60]. As for gender, more male participants were recruited and male patients were found to have a better recovery than female patients, which was consistent with the evidence that the prevalence rate of stroke was higher among men than women in the Chinese population [49], but the female was associated with prolonged dysphagia and increased death [30, 59, 61]. Increased age was shown to have poorer recovery from dysphagia, which was consistent with the advocate that the elder had reduced cognitive function and decreased ability to compensate for changes in skeletal muscle strength, and these factors could affect the outcome of post-stroke swallowing impairment [29, 50, 51, 61–64].

## Limits and Outlook

Despite this study providing promising insights into dysphagia incidence and potential risk factors following the cerebellum, there are some limitations in this study related to its retrospective design. First, among the subjects with dysphagia resulting from cerebellar stroke included in this study between 2014 and 2021, some of them participated in national research on swallowing-related topics; their swallowing function assessment (including swallowing bedside examination, VFSS, and FEES, etc.) before and after treatment during hospitalization were recorded. However, the patients with routine in-patient healthcare for dysphagia mostly receive swallowing function assessment at admission, and these patients after discharge generally seldom participate in swallowing instrumenting examinations voluntarily. The SLP’s bedside examination and the results of the Kubota water test were the only assessment data that existed. At the same time, the main complaints of the patient or his family are used as the basis for judging the treatment effect after discharge and follow-up time. These may affect the accuracy of the judgment on the recovery of dysphagia after cerebellar stroke. The presence of swallowing difficulties after discharge is reported by the patients themselves and their family members, which may influence the accuracy of the prognosis of dysphagia following a cerebellar stroke. Second, patients who had acute medical care for the first onset of a cerebellar stroke in other hospitals, with records of dysphagia or intervention for dysphagia, or who wore a nasogastric tube on admission to this hospital were also included in this study. For this population, we could only judge the presence of dysphagia before admission by reviewing medical records, and inquiring about the patient and their family member, which might lead to judgment bias without the results of VFSS/FEES. Moreover, patients who did not cough during the Kubota water swallowing test would not be referred to an SLP for VFSS/FESS, but they might have a possibility of silent aspiration. Therefore, dysphagia following a cerebellar stroke might be underdiagnosed. Third, cases with the cognitive disorder were not included in the current study so the possible impact of cognitive function on swallowing function cannot be identified. Evidence advocated that the cerebellum is involved in motor, cognition, and mood, and impaired motor, cognitive, and mood function resulting from injuries to the cerebellum could all affect the voluntary process of swallowing [7, 10]. Thus, further studies with a prospective design will provide all the participants only with VFSS or FEES, and will consider the influence of motor, cognition, and mood on the occurrence of dysphagia following isolated cerebellar strokes.

## Conclusion

This study provides preliminary information regarding dysphagia incidence following cerebellar stroke, and factors that may induce swallowing difficulty and influence clinical recovery. Dysphagia can affect a substantial portion of patients in this population, and is associated with patients who have both ischemic and hemorrhagic strokes, multiple lesions in the cerebellum, and increasing age. The recovery rate of the lesions in the cerebellum from best to worst was the right cerebellum, vermis or peduncle, bilateral cerebellar hemisphere, and left cerebellum. This information provides insight into the role of the cerebellum in the process of deglutition and enhances awareness of those individuals most at risk of dysphagia and poor clinical outcome.

## Data Availability

Raw data were generated at Shanghai Yueyang Hospital of Integrated Traditional Chinese and Western Medicine affiliated to Shanghai University of Traditional Chinese Medicine. Derived data supporting the findings of this study are available from the corresponding author upon reasonable request.
